# Supplementary material for: Identification of photoperiod-induced specific miRNAs in the adrenal glands of Sunite sheep (Ovis aries)
Source: Front Vet Sci. 2022 Jul 22;9:888207. doi: 10.3389/fvets.2022.888207 (PMC9354845; doi:10.3389/fvets.2022.888207)
Supplement: Supplementary file 1 [file Data_Sheet_1.ZIP › Supplementary file/miRDeep2 file.zip!/miRDeep2 file/Novel_Structure/SP42A3/Novel_477.pdf]

Provisional ID	:	NC_019484.2_32270
Score total	:	1.6
Score for star read(s)	:	-1.3
Score for read counts	:	-0.8
Score for mfe	:	2.2
Score for randfold	:	1.6
Score for cons. seed	:	
Total read count	:	10
Mature read count	:	3
Loop read count	:	0
Star read count	:	7

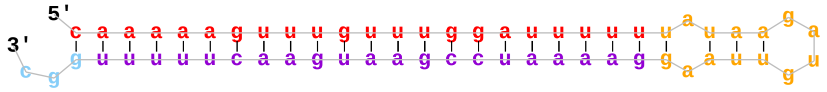

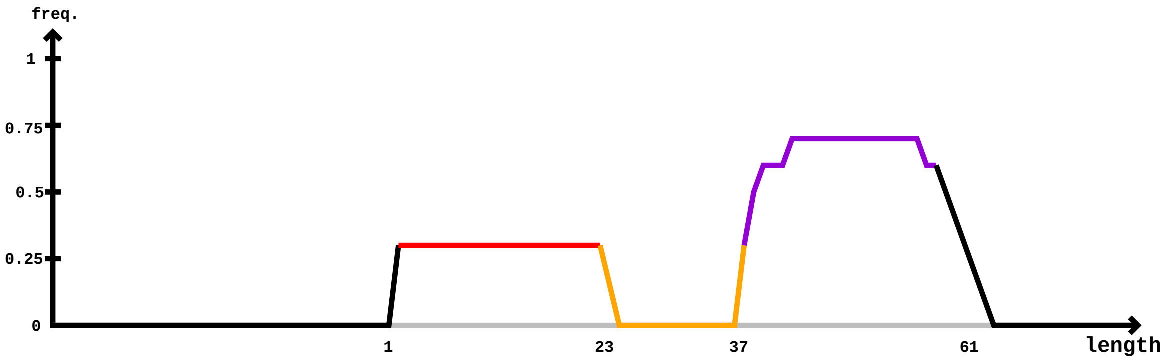

Mature Star

```
5'- uacaugugcgaauuguuacuaauauugggcuggccaaaaaguuuguuuggauuuuuuauaagauguuaaggaaaauccgaaugaacuuuuuggccugcccaauacaucugc   -3'    obs
    uacaugugcgaauuguuacuaauauugggcuggccaaaaaguuuguuuggauuuuuuauaagauguuaaggaaaauccgaaugaacuuuuuggccugcccaauacaucugc          exp
    ......................((((((((.((((((((((((((((((((((((((.(((....))).)))))))))))))))))))))))))).))))))))).......    reads       mm          sample
    ..................................caaaaaguuuguuuggGuuuuu.......................................................    2           1           seq
    ..................................caaaaaguuuguuuggGuuuuuu......................................................    1           1           seq
    ......................................................................gaaaaucUgaaugaacuuu......................    1           1           seq
    ......................................................................gaaaaucUgaaugaacuuuuu....................    1           1           seq
    ......................................................................gaaaauccgaaugaacuuuuu....................    1           0           seq
    .......................................................................aaaauccgaaugaacuuuuug...................    1           0           seq
    .......................................................................aaaauccgaaugaacuuuuuggU.................    1           1           seq
    ........................................................................aaauccgaaugaacuuuuugC..................    1           1           seq
    ...........................................................................uccgaaugaacuuuuuggccu...............    1           0           seq
```
